# The Association between Genetic Variations of *CHI3L1*, Levels of the Encoded Glycoprotein YKL-40 and the Lipid Profile in a Danish Population

**DOI:** 10.1371/journal.pone.0047094

**Published:** 2012-10-05

**Authors:** Stine Brinkløv Thomsen, Camilla Noelle Rathcke, Tea Skaaby, Allan Linneberg, Henrik Vestergaard

**Affiliations:** 1 Center of Endocrinology and Metabolism, Dept. of Medicine, Copenhagen University Hospital, Herlev, Denmark; 2 Department of Cardiology, Nephrology and Endocrinology, Copenhagen University Hospital Hillerød, Denmark; 3 Research Centre for Prevention and Health, Copenhagen University Hospital, Glostrup, the Capital Region of Denmark; 4 Faculty of Health Sciences, University of Copenhagen, Denmark; Innsbruck Medical University, Austria

## Abstract

**Background:**

The inflammatory biomarker YKL-40 seems to play a role in atherosclerosis and is elevated in patients with obesity, cardiovascular disease and type 2 diabetes. Single nucleotide polymorphisms (SNPs) of the YKL-40 encoding gene, *CHI3L1*, are associated with inter-individual YKL-40 levels. One study has described an association between a promoter polymorphism of *CHI3L1* and levels of low density lipoprotein. The objective of this study was to evaluate the influence of YKL-40 on lipid parameters by determining the association between polymorphisms of *CHI3L1*, serum YKL-40 and levels of the differentiated lipid profile in a Danish general population.

**Methodology/Principle Findings:**

12 SNPs of *CHI3L1* were genotyped, and serum YKL-40 and parameters of the lipid profile were measured in 2,656 Danes. Lipid profile and genotypes were available in another Danish population (n = 6,784) for replication. Cholesterol and triglyceride levels increased with increasing YKL-40 quartile (both p<0.0001), and YKL-40 correlated with triglyceride levels (β = 0.15, p<0.0001). Low density lipoprotein levels increased slightly from the 1^st^ to the 3^rd^ quartile (p = 0.006). The highest YKL-40 quartile was associated with a greater risk of hypercholesterolemia compared to the lowest YKL-40 quartile (odds ratio 1.36, p = 0.009). Minor homozygosity of rs12123883 was associated with higher triglyceride levels (p = 0.022) and a higher prevalence of low high density lipoprotein (p = 0.012), but these associations could not be confirmed in the replication population.

**Conclusions/Significance:**

Serum YKL-40 correlates with triglyceride levels in a representative group of the general Danish population. No consistent associations between SNPs of *CHI3L1* and lipid levels could be documented.

## Introduction

Obesity and dyslipidemia are associated with the development of atherosclerosis[1], cardiovascular disease (CVD)[2], insulin resistance and type 2 diabetes (T2D)[3]. Several studies document that elevated levels of triglyceride and low density lipoprotein (LDL) as well as decreased levels of high density lipoprotein (HDL) are major risk factors for the development of CVD [4]–[6]. Moreover, elevated levels of free fatty acids (FFA) are closely linked to insulin resistance and the development of T2D[7], possibly due to the activation of the innate immune system and the establishment of a low grade inflammatory state[6]–[8]. Morbid obesity is characterized by low grade inflammation with elevated levels of several inflammatory markers[9] , and an increased risk of CVD as well as T2D [10].

The inflammation marker YKL-40, also named chitinase-3-like-1 (*CHI3L1*) is elevated in patients with CVD [11], [12] and in patients with type 1 diabetes (T1D)[13] and T2D[14] . In T2D patients and in patients with stable coronary artery disease (CAD), YKL-40 is correlated with FFA and triglyceride levels [14], [15], and in morbidly obese patients YKL-40 declines after weight loss [16]. However, no significant correlation with BMI [14], [16], [17] has been demonstrated.

Studies of single nucleotide polymorphisms (SNPs) of the YKL-40 encoding gene, *CHI3L1*, have documented that genetic variations of *CHI3L1* influence YKL-40 serum levels in healthy subjects[18], [19] and in patients with various inflammatory diseases[18]–[21]. It has been suggested that polymorphisms of *CHI3L1* could have an impact on the prevalence and severity of CAD [19], but this remains to be fully elucidated. However, a single study has described an association between a promoter polymorphism of *CHI3L1* (rs946261) and LDL levels in a Korean population[22].

The objective of the present study was to investigate the possible influence of YKL-40 on lipid levels, and the association of genetic variations of the *CHI3L1* locus with serum YKL-40 levels and parameters of the lipid profile in two groups of the Danish population.

## Methods

### The MONICA-10 population

From 1982 to 1984 the Danish MONICA 1 population study was conducted at the Research Centre for Prevention and Health, Copenhagen University Hospital, Glostrup. This study was part of the Danish contribution to the international MONICA project (MONItoring trends and determinants of CArdiovascular disease). In total, 4,581 individuals of Danish origin, born in 1923, 1933, 1943 and 1953, were randomly selected from 11 municipalities within Copenhagen County. 3,785 individuals (79%) participated. From June 1993 to December 1994, 4,130 former participants were re-invited for the MONICA-10 study, and of those, 2,656 (64%) accepted. These individuals were now 41–43, 51–53, 61–63 or 71–73 years of age [23]–[25].

All individuals participated in the subsequent clinical examinations, performed in one day for each person, and completed a self-administered questionnaire concerning medical history, intake of medication and lifestyle. A trained nurse retrieved anthropometric measures. Waist-hip ratio was calculated according to WHO guidelines[26]. Mean blood pressure was calculated on the basis of two measurements of arterial blood pressure, measured in the sitting position with a random zero mercury sphygmanometer. Heart rate was counted over 15 seconds and calculated per minute. Insulin resistance was determined by HOMA (http://dtu.ox.ac.uk/homacalculator/index.php).

Fasting blood samples were obtained, and the lipid profile and inflammatory markers were measured as previously described [24], [27]. Serum YKL-40 was analysed using a commercial ELISA assay (Quidel, USA), measuring range 20 to 300 ng/ml. Serum high-sensitive C-reactive protein (hsCRP) was analysed using a particle-enhanced immunoturbidimetry assay (Roche/Hitachi), measuring range 0.1–20 mg/l (lowest detection limit 0.03 mg/l). The lipid profile was measured using enzymatic colorimetric methods (Roche, Mannheim, Germany) [27]. 12 SNPs of *CHI3L1* were genotyped.

### Population for replication studies

Replication of the main findings regarding the association between the *CHI3L1* SNPs and lipid parameters was investigated in another population: the Inter99 population. The study design and characteristics of the participants have previously been described in detail [28]. In brief, an age- and sex-stratified random sample of 13,016 men and women born in 1939–40, 1944–45, 1949–50, 1954–55, 1959–60, 1964–65, 1969–70 and living in 11 municipalities in the South-Western part of the former Copenhagen County was drawn from the Civil Registration System and invited to a health examination. A total of 12,934 persons were eligible for invitation of whom 6,784 (52.5%) participated. In general the participation rate was higher in women than in men, and it increased with increasing age. In addition, non-responders had more hospital admissions related to chronic diseases like diabetes and cardiovascular disease [24]. The examinations of participants in this study were completed from March 1999 through January 2001. The health examination included a self-administered questionnaire, a physical examination, and various blood tests. The lipid profile was measured using enzymatic techniques (Boeringer Mannheim, Germany) [28], and 11 SNPs of *CHI3L1* were already genotyped for a previous study[29]. Only participants with a Northern European origin were included in the current study (n = 6,405). Information on current and former nationalities of participants as well as their parents was obtained from Statistics Denmark and from the self-administered questionnaire. A Northern European origin was defined as a Danish, Norwegian, Swedish, Icelandic, or Faroese nationality. A non-Northern European origin was defined as nationalities other than the above mentioned. Both current and potential former nationalities of participants and their parents were considered.

### Ethics Statement

The MONICA-10 study and the Inter99 study were approved by the Local Ethics Committee of Copenhagen County (KA-04130 and KA 98155, respectively). Both studies were conducted in accordance with the Helsinki Declaration, and all participants gave informed written consent to participation.

### Definitions

Persons smoking one or more cigarettes/cigars/pipes a day were classified as smokers; all others were classified as non-smokers. Hypertension was defined as systolic blood pressure ≥140 mmHg, diastolic blood pressure ≥90 mmHg or use of antihypertensive drugs. Hypercholesterolemia was defined as use of cholesterol lowering drugs or a baseline serum cholesterol level >5 mmol/l. Low HDL was defined as serum HDL <1.0 mmol/l for men and <1.2 mmol/l for women.

### Genotyping of single nucleotide polymorphisms (SNPs) in the CHI3L1 gene

The participants were genotyped for SNPs in the *CHI3L1* gene. A region 22 kb upstream and 10 kb downstream of CHI3L1 was chosen from the HapMap project (www.hapmap.org) and HapMap Data Rel 21a/phasell jan07, on NCBI assembly, dbSNP b125, was used for the SNP selection. For the MONICA-10 population, a total of 12 tgSNPs located in the region 14 kb upstream to 2 kb downstream of CHI3L1, covering all linkage disequilibrium (LD) blocks in CHI3L1, were genotyped. For the Inter99 population, 11 of the 12 SNPs were genotyped. TAGGER[30] chose these SNPs as the most informative in the chosen +22 kb - −10 kb region. TAGGER was used with a 5% minor allele frequency (MAF) cut off and aggressive tagging, i.e. r^2^>0.8.

Genotyping of both the MONICA-10 and Inter99 populations was performed using KBiosciences allele-specific PCR (KASPar) (KBioscience, Herts, UK) with a success rate >97.4%. Genotype distribution obeyed Hardy Weinberg equilibrium (HWE), all p>0.14 using Genepop v4.0.10[31]

### Statistical analyses

Analyses were performed using the statistical software package SPSS 18.0 (SPSS inc., Chicago, Il, USA) for the MONICA-10 population and SAS, version 9.3 (SAS Institute Inc. Cary, NC USA) for the Inter-99 study. P-values were two-sided, and p-values<0.05 were considered statistically significant. Continuous variables were presented as mean (standard deviation (SD)) if they were normally distributed (BMI, WHR, BP, total cholesterol, LDL, HDL) or median (inter quartile range (IQR)) if they had a non-Gaussian distribution (triglyceride, YKL-40, hsCRP). Categorical variables were presented as numbers (%). If data had a non-Gaussian distribution as revealed by P-Plot, data was logarithmically transformed using the natural logarithm before implemented in further statistical analyses.

For the MONICA-10 population the following analyses were performed: All baseline characteristics were analysed and presented according to YKL-40 quartiles. Categorical data were compared using the chi-square test for k independent samples, and continuous data were compared with One-Way ANOVA. Analyses of correlations between YKL-40 and lipid parameters were performed as a backward regression analysis with adjustment for gender, age, alcohol, hypertension, waist-hip ratio, plasma glucose, CRP and insulin resistance, and results were reported as β coefficients with 95% confidence intervals (95% CI). The odds ratios (OR) of hypercholesterolemia and low HDL according to YKL-40-quartiles were determined by logistic regression with adjustment for age and gender. The association between polymorphisms of *CHI3L1* and prevalence of hypercholesterolemia and low HDL was similarly examined by logistic regression analyses with adjustment for age and gender, and presented as OR (95%CI).

For the MONICA-10 and the Inter99 population the association between polymorphisms of *CHI3L1* and parameters of the lipid profile was examined in linear regression models with adjustments for age and gender, where the SNPs were tested as categorical variables. The Bonferroni method was used to adjust for multiple comparisons. The results of the adjusted p-values are reported in the text of the results section.

## Results

Baseline characteristics according to YKL-40 quartiles are presented in Table 1. There were more men and older individuals (71–73 years) in the 4^th^ YKL-40 quartile (p<0.0001 for both). BMI (p<0.0001) and WHR (p<0.0001) were higher in the 3^rd^ and 4^th^ quartile, and total cholesterol (p<0.0001) and triglyceride levels (p<0.0001) increased with each YKL-40 quartile. LDL levels increased slightly from the 1^st^ to the 3^rd^ quartile (p = 0.006), whereas HDL did not change significantly (p = 0.063). The prevalence of hypercholesterolemia increased with each YKL-40 quartile (p<0.0001), and the prevalence of low HDL was highest in the 4^th^ quartile (p = 0.021). Systolic and diastolic blood pressure as well as the prevalence of hypertension and use of BP lowering medicine were higher in the two highest quartiles (p<0.0001 for all). Median levels of hsCRP increased with each YKL-40 quartile (p<0.0001).

**Table 1:** Characteristics at baseline according to YKL-40 quartiles in MONICA-10.

	1^st^ quartile	2^nd^ quartile	3^rd^ quartile	4th quartile	p-value
YKL-40 range	≤40	>40, ≤57	>57, ≤85	>85	
N	677	672	633	660	<0.0001
Male	302 (44.6)	327 (48.7)	316 (49.9)	378 (57.3)	<0.0001
41–43 years	266 (39.3)	224 (33.3)	142 (22.4)	91 (13.8)	
51–53 years	226 (33.4)	201 (29.9)	169 (26.7)	143 (21.7)	
61–63 years	134 (19.8)	165 (24.6)	166 (26.2)	211 (32.0)	
71–73 years	51 (7.5)	82 (12.2)	156 (24.6)	215 (32.6)	<0.0001
BMI, kg/m^2^*	25.2 (3.7)	25.8 (3.9)	26.2 (4.3)	26.7 (4.6)	<0.0001
WHR*	0.86 (0.09)	0.87 (0.08)	0.89 (0.08)	0.91 (0.09)	<0.0001
Systolic BP, mmHg*	125 (17)	126 (18)	131 (20)	136 (19)	<0.0001
Diastolic BP, mmHg*	80 (10)	81 (10)	83 (11)	84 (11)	<0.0001
Hypertension	220 (32.5)	225 (33.5)	275 (43.4)	352 (53.3)	<0.0001
BP lowering medicine	64 (9.5)	52 (7.7)	101 (16.0)	124 (18.8)	<0.0001
Cholesterol, mmol/l*	6.0 (1.0)	6.1 (1.1)	6.2 (1.1)	6.4 (1.1)	<0.0001
LDL, mmol/l*	3.9 (1.0)	4.0 (1.0)	4.1 (1.0)	4.1 (1.0)	0.006
HDL, mmol/l*	1.5 (0.4)	1.4 (0.4)	1.4 (0.4)	1.5 (0.5)	0.063
Triglycerides, mmol/l**	1.0 (0.8–1.4)	1.1 (0.8–1.6)	1.3 (0.9–1.8)	1.5 (1.0–2.2)	<0.0001
Low HDL	59 (8.7)	82 (12.2)	81 (12.8)	92 (13.9)	0.021
Hypercholesterolemia	311 (45.9)	340 (50.6)	345 (54.6)	413 (62.6)	<0.0001
hsCRP, mg/l**	1.2 (0.6–2.5)	1.4 (0.7–3.1)	2.2 (1.0–4.4)	2.8 (1.4–5.9)	<0.0001

Values are presented as *mean (SD), **median (IQR) or N (% within quartile) where not specified.

Abbreviations: BMI, body mass index; WHR, waist-hip-ratio; BP, blood pressure; LDL, low density lipoprotein; HDL, high density lipoprotein; hsCRP, high sensity C-reactive protein; SD, standard deviation; IQR, inter quartile range.

Linear regression models with YKL-40 as the independent factor and lipids, BMI or WHR as the dependent factors were performed to determine the possible link between YKL-40 levels and the development of dyslipidaemia and obesity. YKL-40 correlated with triglyceride levels (β = 0.15, p<0.0001) after adjustment for gender, age, alcohol, pulse, hypertension, waist-hip ratio, plasma glucose, hsCRP and insulin resistance (all determinants of YKL-40 with R-values ≥0.15). Weaker correlations of YKL-40 with total cholesterol (β = 0.10, p = 0.008) and HDL (β = 0.04, p = 0.001) were found. There was no significant correlation with LDL.

YKL-40 correlated positively with BMI, when adjusting for age and gender (β = 0.45, p = 0.001). However, when adjusting for gender, age, alcohol, pulse, hypertension, waist-hip ratio, plasma glucose, hsCRP and insulin resistance, we found a negative correlation instead (β = −0.34, p = 0.009). YKL-40 did not correlate with WHR. However, there was a greater risk of hypercholesterolemia within the 4^th^ YKL-quartile compared to the 1^st^ YKL-40 quartile (OR 1.36, p = 0.009) after adjustment for age and gender.

### Single nucleotide polymorphisms of CHI3L1

Table 2 presents the relationship between 12 SNPs of *CHI3L1* and levels of lipid parameters and YKL-40 for the MONICA-10 population. P-values are derived from linear regression models with adjustments for age and gender. All SNPs presented MAFs >5% except for rs4950930 (MAF = 4.7%). Rs12123883 genotypes presented statistically significant differences in triglyceride levels. A median triglyceride value of 1.7 (IQR 1.2; 2.3) mmol/l was seen with minor allele homozygosity, compared to 1.2 (IQR 0.9; 1.7) and 1.1 (IQR 0.9; 1.6) mmol/l for major allele homozygosity and heterozygosity, respectively (p = 0.022). This SNP also presented a significantly higher YKL-40 level with minor allele homozygosity (84 [IQR 55; 233] ng/ml) compared to major allele homozygosity (56 [IQR 40; 84] ng/ml) and heterozygosity (58 [IQR 43; 86] ng/ml) (p = 0.001). Rs872129 presented with a significantly higher level of total cholesterol for minor allele homozygosity with a mean value of 6.7 (SD 1.1) mmol/l, compared to 6.1 (SD 1.1) mmol/l for both of the other genotypes (p = 0.021). Triglyceride levels were also higher in minor allele homozygote with a median value of 1.3 (IQR 1.1;1.6) mmol/l, compared to 1.2 (IQR 0.9;1.7) mmol/l for major allele homozygote and 1.2 (IQR0.8;1.7) mmol/l for allele heterozygote, respectively (p = 0.022). However, this SNP presented with the highest YKL-40 level with allele heterozygosity (64 [IQR 45; 100] ng/ml) compared to minor allele homozygosity (60 [IQR 47; 112] ng/ml) and major allele homozygosity (55 [IQR 39; 82] ng/ml), respectively (p<0.0001). None of the remaining SNPs presented with significant differences in allele-associated lipid levels. Except for one SNP (rs4950930) all of the remaining SNPs showed statistically significant differences in YKL-40 levels between genotypes (p<0.0001 for all). When adjusting for multiple comparisons by the Bonferroni method, only the p-values from analyses regarding YKL-40 remained statistically significant, except for rs12123883 (p = 0.06).

**Table 2:** The relationship between 12 single nucleotide polymorphisms (SNPs) of *CHI3L1* and levels of lipid parameters and YKL-40 in MONICA-10.

SNP	Genotype distribution		Cholesterol, mmol/l		LDL, mmol/l		HDL, mmol/l		Triglyceride, mmol/l		YKL-40, ng/ml	
	All	Prevalence, n (%)	mean (SD)	p-value**	mean (SD)	p-value**	mean (SD)	p-value**	median (IQR)	p-value**	median (IQR)	p-value**
rs10399931	CC *	1478 (57.9)	6.2 (1.1)	0.880	4.0 (1.0)	0.811	1.5 (0.4)	0.968	1.2 (0.9–1.7)	0.680	66 (49–97)	**<0.0001**
	CT	933 (36.6)	6.2 (1.1)		4.0 (1.0)		1.4 (0.4)		1.2 (1.0–1.8)		47 (35–68)	
	TT	141 (5.5)	6.1 (1.1)		4.0 (1.0)		1.4 (0.4)		1.2 (0.9–1.7)		27 (20–40)	
rs12123883	TT *	2165 (84.3)	6.2 (1.1)	0.609	4.0 (1.0)	0.948	1.4 (0.4)	0.470	1.2 (0.9–1.7)	**0.022**	56 (40–84)	**0.001**
	TC	391 (15.2)	6.1 (1.1)		4.0 (1.0)		1.5 (0.4)		1.1 (0.9–1.6)		58 (43–86)	
	CC	13 (0.5)	6.2 (1.6)		3.8 (0.8)		1.2 (0.4)		1.7 (1.2–2.3)		84 (55–233)	
rs2486064	GG *	811 (31.8)	6.2 (1.2)	0.631	4.1 (1.3)	0.760	1.5 (0.4)	0.303	1.2 (0.9–1.6)	0.229	65 (48–97)	**<0.0001**
	GA	1247 (49.0)	6.1 (1.1)		4.0 (1.0)		1.4 (0.4)		1.2 (0.9–1.8)		55 (39–84)	
	AA	490 (19.2)	6.2 (1.1)		4.0 (1.0)		1.5 (0.4)		1.2 (0.9–1.7)		44 (31–67)	
rs2886117	GG *	1944 (76.9)	6.1 (1.1)	0.051	4.0 (1.0)	0.080	1.4 (0.4)	0.299	1.2 (0.9–1.7	0.524	54 (38–80)	**<0.0001**
	GA	542 (21.5)	6.2 (1.1)		4.1 (1.1)		1.5 (0.4)		1.2 (0.9-1.7)		65 (47–99)	
	AA	41 (1.6)	6.4 (0.9)		4.2 (0.9)		1.5 (0.5)		1.1 (0.9–1.6)		72 (51–120)	
rs4950928	CC *	1592 (62.2)	6.2 (1.1)	0.670	4.0 (1.0)	0.664	1.4 (0.4)	0.865	1.2 (0.9–1.7)	0.476	66 (49–97)	**<0.0001**
	CG	854 (33.4)	6.2 (1.1)		4.0 (1.0)		1.4 (0.4)		1.2 (0.9–1.8)		45 (34–64)	
	GG	113 (4.4)	6.2 (1.2)		4.1 (1.0)		1.4 (0.4)		1.2 (1.0–1.8)		26 (19–35)	
rs4950930	GG *	2319 (90.9)	6.2 (1.1)	0.781	4.0 (1.0)	0.814	1.4 (0.4)	0.944	1.2 (0.9–1.7)	0.795	56 (40–84)	0.074
	GA	225 (8.8)	6.1 (1.2)		4.1 (1.1)		1.4 (0.4)		1.2 (0.9–1.6)		65 (45–93)	
	AA	7 (0.3)	6.4 (1.1)		4.2 (1.1)		1.4 (0.4)		1.1 (1.0–3.3)		56 (46–84)	
rs6691378	GG *	2001 (78.4)	6.1 (1.1)	0.196	4.0 (1.0)	0.305	1.4 (0.4)	0.491	1.2 (0.9–1.7)	0.745	54 (39–80)	**<0.0001**
	GA	518 (20.3)	6.2 (1.2)		4.1 (1.1)		1.5 (0.4)		1.2 (0.9–1.7)		66 (47–100)	
	AA	33 (1.3)	6.4 (0.9)		4.1 (0.8)		1.6 (0.5)		1.1 (0.8–1.6)		81 (53–120)	
rs871799	GG *	2076 (81.2)	6.2 (1.1)	0.773	4.0 (1.0)	0.936	1.4 (0.4)	0.213	1.2 (0.9–1.7)	0.328	55 (39–82)	**<0.0001**
	GC	448 (17.5)	6.2 (1.1)		4.0 (1.0)		1.5 (0.4)		1.2 (0.9–1.6)		64 (45–96)	
	CC	32 (1.3)	6.1 (1.0)		4.0 (0.9)		1.4 (0.3)		1.3 (0.9–1.8)		67 (49–110)	
rs872129	AA *	2160 (84.4)	6.1 (1.1)	**0.021**	4.1 (1.0)	0.238	1.4 (0.4)	0.713	1.2 (0.9–1.7	**0.022**	55 (39–82)	**<0.0001**
	AG	386 (15.1)	6.1 (1.1)		4.0 (1.0)		1.5 (0.4)		1.2 (0.8–1.7)		64 (45–100)	
	GG	12 (0.5)	6.7 (1.1)		4.5 (1.0)		1.4 (0.3)		1.3 (1.1–1.6)		60 (47–112)	
rs880633	CC *	734 (28.7)	6.1 (1.1)	0.435	4.0 (1.0)	0.302	1.4 (0.4)	0.243	1.2 (0.9–1.7)	0.902	62 (47–89)	**<0.0001**
	CT	1284 (50.3)	6.2 (1.1)		4.1 (1.0)		1.4 (0.4)		1.2 (0.9–1.7)		56 (40–86)	
	TT	536 (21.0)	6.2 (1.1)		4.1 (1.0)		1.5 (0.4)		1.2 (0.9–1.8)		49 (32–75)	
rs883125	CC *	1842 (71.6)	6.2 (1.1)	0.181	4.0 (1.0)	0.072	1.4 (0.4)	0.118	1.2 (0.9–1.7)	0.283	53 (38–80)	**<0.0001**
	CG	673 (26.2)	6.2 (1.1)		4.0 (1.0)		1.5 (0.4)		1.2 (0.9–1.7)		64 (45–98)	
	GG	56 (2.2)	6.2 (1.2)		4.0 (1.1)		1.5 (0.5)		1.2 (0.8–1.5)		63 (49–86)	
rs946263	AA *	1647 (64.6)	6.2 (1.1)	0.649	4.0 (1.1)	0.714	1.4 (0.4)	0.715	1.2 (0.9–1.7)	0.437	65 (47–97)	**<0.0001**
	AG	812 (31.8)	6.2 (1.1)		4.0 (1.0)		1.4 (0.4)		1.2 (0.9–1.8)		45 (33–64)	
	GG	92 (3.6)	6.3 (1.2)		4.2 (1.0)		1.5 (0.4)		1.2 (0.9–1.7)		26 (19–36)	

* major allele ** Linear regression models, adjusted for age and gender. All SNPs presented MAFs (minor allele frequenciy)>5%, except for rs4950930 (MAF = 4.7%)

Abbreviations: LDL, low density lipoprotein; HDL, high density lipoprotein.

Correlations between SNPs of *CHI3L1* and lipid levels were examined in linear regression models with adjustments for age and gender. Rs2886117 was weakly correlated with total cholesterol levels (β = −0.11 change per allele, p = 0.020) and LDL levels (β = −0.09 change per allele, p = 0.044). None of the other SNPs correlated with the lipid parameters.

Table 3 presents the prevalence and risk of hypercholesterolemia and low HDL according to the 12 SNPs of *CHI3L1* for the MONICA-10 population. Minor allele homozygosity of rs12123883 (p = 0.012) and major allele homozygosity of rs872129 (p = 0.05) were associated with a higher prevalence of low HDL compared to the other genotypes.

**Table 3:** Prevalence and risk (odds ratio (95% confidence interval)) of hypercholesterolemia and low HDL at baseline according to single nucleotide polymorphisms (SNPs) of CHI3L1 in MONICA-10.

SNP	genotype	Hypercholesterolemia		low HDL	
		prevalence	OR (95% CI)	prevalence	OR (95 CI)
rs10399931	CC*	53.2 (787/1478)	1	12.2 (181/1478)	1
	CT	54.3 (507/933)	1.05 (0.88–1.24)	11.6 (108/933)	0.94 (0.72–1.21)
	TT	48.2 (68/141)	0.74 (0.52–1.06)	8.5 (12/141)	0.66 (0.35–1.23)
		p = 0.394	p = 0.176	p = 0.408	p = 0.374
rs12123883	TT *	53.4 (1156/2165)	1	11.7 (254/2165)	1
	TC	52.2 (204/391)	1.00 (0.88–1.22)	11.5 (45/391)	0.97 (0.69–1.37)
	CC	46.2 (6/13)	0.70 (0.22–2.2)	38.5 (5/13)	4.06 (1.26–13.11)
		p = 0.796	p = 0.829	**p = 0.012**	p = 0.091
rs2486064	GG*	53.1 (431/811)	1	11.0 (89/811)	1
	GA	53.7 (670–1247)	1.03 (0.86–1.22)	12.9 (161/1247)	1.21 (0.91–1.60)
	AA	52.2 (256/490)	0.94 (0.74–1.18)	10.6 (52/490)	0.93 (0.64–1.34)
		p = 0.853	p = 0.685	p = 0.265	p = 0.205
rs2886117	GG*	63.4 (26/41)	1	11.8 (229/1944)	1
	GA	56.3 (305–542)	1.20 (0.98–1.46)	12.7 (69/542)	1.12 (0.83–1.50)
	AA	52.3 (1016–1944)	1.47 (0.76–2.85)	4.9 (2/41)	0.42 (1.00–1.79)
		p = 0.108	p = 0.115	p = 0.314	p = 0.296
rs4950928	CC*	53.0 (843/1592)	1	11.7 (187/1592)	1
	CG	53.2 (454/854)	1.00 (0.84–1.19)	12.1 (103/854)	1.06 (0.81–1.37)
	GG	54.0 (61/113)	0.91 (0.61–1.35)	8.8 (10/113)	0.74 (0.37–1.46)
		p = 0.976	p = 0.895	p = 0.608	p = 0.575
rs4950930	GG*	53.4 (1239/2319)	1	11.8 (273/2319)	1
	GA	50.2 (113–225)	0.86 (0.64–1.14)	11.1 (25/225)	0.89 (0.57–1.38)
	AA	57.1 (4/7)	1.38 (0.30–6.35)	28.6 (2/7)	2.41 (0.44–13.12)
		p = 0.640	p = 0.507	p = 0.368	p = 0.538
rs6691378	GG*	52.1 (1042–2001)	1	11.8 (236/2001)	1
	GA	55.4 (287/518)	1.17 (0.95–1.43)	12.5 (65/518)	1.10 (0.82–1.49)
	AA	66.7 (22/33)	1.75 (0.83–3.72)	6.1 (2/33)	0.50 (0.12–2.16)
		p = 0.113	p = 0.122	p = 0.521	p = 0.471
rs871799	GG*	52.8 (1096/2076)	1	12.4 (257/2076)	1
	GC	54.7 (245/448)	1.05 (0.85–1.30)	10.0 (45/448)	0.80 (0.57–1.12)
	CC	43.8 (14/32)	0.66 (0.32–1.36)	6.3 (2/32)	0.44 (0.10–1.89)
		p = 0.439	p = 0.465	p = 0.234	p = 0.207
rs872129	AA*	53.5 (1156/2160)	1	12.5 (271/2160)	1
	AG	51.0 (197/386)	0.91 (0.73–1.14)	8.8 (34/386)	0.67 (0.45–0.97)
	GG	75.0 (9/12)	2.34 (0.62–8.87)	0 (0/12)	0.00 (0.00-)
		p = 0.212	p = 0.290	**p = 0.050**	**p = 0.026**
rs880633	CC*	51.1 (375/734)	1	12.1 (89/734)	1
	CT	54.1 (695/1284)	1.12 (0.93–1.35)	12.4 (159/1284	1.00 (0.75–1.32)
	TT	53.9 (289/536)	1.09 (0.87–1.37)	10.1 (54/536)	0.80 (0.56–1.16)
		p = 0.393	p = 0.491	p = 0.364	p = 0.394
rs883125	CC*	52.6 (968/1842)	1	11.8 (217/1842)	1
	CG	53.9 (363/673)	1.06 (0.88–1.27)	12.0 (81/673)	0.98 (0.74–1.29)
	GG	60.7 (34/56)	1.43 (0.82–2.51)	10.7 (6/56)	0.87 (0.36–2.10)
		p = 0.424	p = 0.399	p = 0.952	p = 0.942
rs946263	AA*	52.9 (871/1647)	1	11.5 (189/1647)	1
	AG	53.8 (437/812)	1.03 (0.86–1.22)	12.7 (103/812)	1.15 (0.87–1.50)
	GG	58.7 (54/92)	1.06 (0.68–1.64)	8.7 (8/92)	0.74 (0.35–1.58)
		p = 0.53	p = 0.942	p = 0.443	p = 0.356

* Major allele

Adjusted for age and gender

Table 4 presents the 11 SNPs of *CHI3L1* for the Inter99 population and the associated levels of the lipid parameters (rs946263 was not analysed). In this population, none of the SNPs presented with statistically significant differences in triglyceride levels. However, rs872129 presented with the lowest values of total cholesterol in minor allele homozygosity (5.1 [SD 0.8] mmol/l) compared to 5.6 (SD 1.1) mmol/l for major allele homozygote and 5.5 (SD1.1) mmol/l for allele heterozygote,(p = 0.048). Rs871799 also presented with statistical differences in levels of total cholesterol with the lowest value in homozygote for the minor allele (5.2 [SD 1.0] mmol/l) compared to 5.6 (SD 1.1) mmol/l for major allele homozygote and 5.5 (SD 1.1) mmol/l for allele heterozygote (p = 0.037.). Rs871799 had the lowest values of LDL in the minor allele homozygote (3.2 (SD 0.9) mmol/l) compared to 3.5 (SD 1.0) mmol/l and 3.5 (SD 0.9) mmol/l for major allele homozygosity and allele heterozygosity (p = 0.022). When adjusting for multiple comparisons by the Bonferroni method, none of the p-values remained statistically significant.

**Table 4:** The relationship between 11 single nucleotide polymorphisms (SNPs) of CHI3L1 and lipid levels in Inter99.

SNP	Genotype distribution		Cholesterol, mmol/l		LDL, mmol/l		HDL, mmol/l		Triglyceride, mmol/l	
	All	Prevalence, n (%)	mean (SD)	p-value**	mean (SD)	p-value**	mean (SD)	p-value**	median (IQR)	p-value**
rs10399931	CC *	3572 (58.5)	5.5 (1.1)	0.867	3.5 (1.0)	0.626	1.4 (0.4)	0.608	1.1 (0.8–1.6)	0.835
	CT	2182 (35.8)	5.5 (1.1)		3.5 (1.0)		1.4 (0.4)		1.1 (0.8–1.6)	
	TT	349 (5.7)	5.6 (1.1)		3.5 (1.0)		1.4 (0.4)		1.1 (0.8–1.6)	
rs12123883	TT *	5296 (86.1)	5.5 (1.1)	0.757	3.5 (1.0)	0.852	1.4 (0.4)	0.395	1.1 (0.8–1.6)	0.802
	TC	816 (13.3)	5.5 (1.1)		3.5 (1.0)		1.4 (0.4)		1.1 (0.7–1.5)	
	CC	40 (0.6)	5.3 (1.2)		3.4 (1.0)		1.3 (0.3)		1.1 (0.8–1.5)	
rs2486064	GG *	2061 (33.6)	5.6 (1.1)	0.333	3.5 (1.0)	0.275	1.4 (0.4)	0.220	1.1 (0.8–1.5)	0.836
	GA	2962 (48.3)	5.5 (1.1)		3.5 (1.0)		1.4 (0.4)		1.1 (0.8–1.6)	
	AA	1109 (18.1)	5.5 (1.1)		3.5 (1.0)		1.4 (0.4)		1.1 (0.8–1.6)	
rs2886117	GG *	4716 (76.5)	5.5 (1.1)	0.929	3.51 (1.0)	0.915	1.4 (0.4)	0.371	1.1 (0.8–1.6)	0.548
	GA	1343 (21.8)	5.6 (1.1)		3.53 (1.0)		1.4 (0.4)		1.1 (0.8–1.6)	
	AA	104 (1.7)	5.6 (1.4)		3.48 (1.0)		1.4 (0.4)		1.1 (0.8–1.5)	
rs4950928	CC *	3899 (63.3)	5.5 (1.1)	0.597	3.5 (1.0)	0.294	1.4 (0.4)	0.704	1.1 (0.8–1.6)	0.943
	CG	1999 (32.5)	5.5 (1.1)		3.5 (1.0)		1.4 (0.4)		1.1 (0.8–1.6)	
	GG	260 (4.2)	5.6 (1.1)		3.6 (1.0)		1.4 (0.4)		1.1 (0.8–1.6)	
rs4950930	GG *	5622 (91.8)	5.5 (1.1)	0.869	3.5 (1.0)	0.696	1.4 (0.4)	0.911	1.1 (0.8–1.6)	0.889
	GA	487 (8.0)	5.5 (1.0)		3.5 (0.9)		1.4 (0.4)		1.1 (0.8–1.6)	
	AA	15 (0.2)	5.5 (1.1)		3.4 (1.1)		1.5 (0.5)		1.1 (0.8–1.8)	
rs6691378	GG *	4760 (77.6)	5.5 (1.1)	0.634	3.5 (1.0)	0.836	1.4 (0.4)	0.715	1.1 (0.8–1.6)	0.776
	GA	1287 (21.0)	5.6 (1.2)		3.5 (1.0)		1.4 (0.4)		1.1 (0.8–1.6)	
	AA	86 (1.4)	5.6 (1.5)		3.5 (1.0)		1.4 (0.5)		1.1 (0.8–1.4)	
rs871799	GG *	4995 (81.3)	5.6 (1.1)	**0.037**	3.5 (1.0)	**0.022**	1.4 (0.4)	0.468	1.1 (0.8–1.6)	0.559
	GC	1084 (17.6)	5.5 (1.1)		3.5 (0.9)		1.4 (0.4)		1.1 (0.8–1.5)	
	CC	67 (1.1)	5.2 (1.0)		3.2 (0.9)		1.4 (0.4)		1.1 (0.8–1.6)	
rs872129	AA *	5212 (84.9)	5.6 (1.1)	**0.048**	3.5 (1.0)	0.121	1.4 (0.4)	0.383	1.1 (0.8–1.6)	0.919
	AG	886 (14.4)	5.5 (1.1)		3.5 (0.9)		1.4 (0.4)		1.1 (0.8–1.5)	
	GG	40 (0.7)	5.1 (0.8)		3.1 (0.7)		1.4 (0.4)		1.1 (0.7–1.4)	
rs880633	CC *	1807 (29.7)	5.5 (1.1)	0.870	3.5 (1.0)	0.842	1.4 (0.4)	0.999	1.1 (0.8–1.6)	0.832
	CT	2995 (49.2)	5.5 (1.1)		3.5 (0.9)		1.4 (0.4)		1.1 (0.8–1.5)	
	TT	1282 (21.1)	5.6 (1.1)		3.5 (1.0)		1.4 (0.4)		1.1 (0.8–1.6)	
rs883125	CC *	4397 (71.8)	5.6 (1.1)	0.239	3.5 (1.0)	0.392	1.4 (0.4)	0.987	1.1 (0.8–1.6)	0.240
	CG	1577 (25.7)	5.5 (1.1)		3.5 (1.0)		1.4 (0.4)		1.1 (0.8–1.6)	
	GG	153 (2.5)	5.6 (1.2)		3.6 (1.1)		1.4 (0.5)		1.2 (0.9–1.5)	

*major allele **Linear regression models, adjusted for age and gender.

Abbreviations: LDL, low density lipoprotein; HDL, high density lipoprotein; SD, standard deviation; IQR, inter quartile range.

## Discussion

This population study is the first of this size to investigate the associations between SNPs of the YKL-40 encoding gene *CHI3L1*, levels of the encoded glycoprotein YKL-40 and the differentiated lipid profile. In 2,656 adult Danes we documented that increasing YKL-40 levels were associated with increasing levels of total cholesterol and triglycerides as well as a higher prevalence of hypercholesterolemia and low HDL. We also found a positive correlation of YKL-40 with triglyceride as well as with total cholesterol levels and a higher risk of hypercholesterolemia within the 4^th^ YKL-40 quartile. A positive correlation was also found between YKL-40 and HDL (β = 0.04, p = 0.001); however, the low β-value makes it difficult to draw any conclusions from this result, and it is interpreted as a chance finding. We did not find a correlation of YKL-40 with LDL levels.

Obesity and dyslipidemia are known risk factors of atherosclerosis[1], CVD[32], [33] and T2D[10], possibly through the initiation of inflammatory processes. The documentation of elevated YKL-40 levels in patients with CVD[11], [12] and T2D[11] as well as the observation of higher YKL-40 levels in patients with obesity[16] has nourished our hypothesis that YKL-40 might be involved in the development of dyslipidemia. In the very first study documenting elevated YKL-40 levels in patients with T2D, we also described an association of YKL-40 with levels of triglycerides and FFA[14] . Previously, it has been documented that a high-fat meal induces systemic low-grade inflammation, triggered by bacterial lipopolysaccharide (LPS) from the gut microbiota, which, in turn, leads to lipolysis[34], [35], perhaps partly mediated by the pro-inflammatory cytokine Interleukin 6[36]. It has been suggested that the LPS-induced lipolysis could reinforce the expression of inflammatory cytokines, creating a ‘vicious circle’ towards low-grade inflammation, dyslipidemia and atherosclerosis[37]. YKL-40 has been shown to initiate the mitogen-activated protein kinase (MAPK) and phosphoinoside-3-kinase (PI3K) signaling pathways in fibroblasts[38], and we speculate if YKL-40 could somehow have a lipolytic effect, directly or indirectly, on the adipocytes, and thereby increasing the plasma levels of the atherogenic lipids.

YKL-40 is considered a marker of inflammation and endothelial dysfunction [39], [40], with a possible function in the development of atherosclerosis[41]. However, the role of YKL-40 in atherosclerosis and atherosclerotic plaque formation is not clear. YKL-40 is thought to be important in the activation of monocytes and their formation into macrophages [42], [43], and to facilitate their transformation into lipid laden foam cells, thereby initiating the first step of plaque-formation[41]. Michelsen et al found that serum YKL-40 levels were significantly elevated in patients with carotid atherosclerosis, with particularly high levels in those with symptomatic disease [44]. *In vitro* studies documented that stimulation with YKL-40 increased matrix metalloproteinase-9 levels in THP-1 monocytes and in peripheral monocytes from healthy donors [44]. This suggests that YKL-40 could be considered a marker of plaque instability, perhaps reflecting the activation of macrophages and matrix degeneration within the atherosclerotic plaque [44].

Previously, a positive association between YKL-40 levels and triglyceride levels has been reported in participants of the Copenhagen City Heart Study. In that study, consisting of 8,899 Danes representative of the general Danish population[45], an increased risk of ischemic stroke and ischemic cerebrovascular disease was found with elevated levels of YKL-40. However, in contrast to our findings, the study did not find higher levels of total cholesterol with the highest YKL-40 levels [45].

Other studies have documented positive correlations between YKL-40 and triglyceride levels in patients with T2D and stable coronary artery disease[14], [15], [46], and we have previously documented a strong association of YKL-40 with total cholesterol and LDL levels in patients with T2D[46]. The last-mentioned study included 105 patients with T2D, of whom more than half had various degrees of kidney disease. These patients may therefore have had a larger burden of low grade inflammation compared to healthy individuals, and may not be directly comparable to the participants in the present study[46]. In another study of 200 patients with various degrees of CAD, Kucur et al found a positive association between YKL-40 levels and the number of diseased vessels. There was no difference between groups regarding levels of triglycerides, cholesterol, LDL or HDL, and no correlation between lipids and YKL-40 levels[47]. Though it is not described, it could be expected that most of these patients were treated with lipid lowering drugs. Moreover, the number of participants was rather small compared to our study, which also makes comparisons of the results difficult. However, the lack of a positive association between plasma YKL-40 levels and lipids could also mean that YKL-40 plays a role in the development of atherosclerosis independently of dyslipidaemia, which is in contrast to our hypothesis.

We found a significant increase of BMI and WHR with increasing YKL-40 quartiles, and BMI was found to correlate positively with YKL-40 when adjusting for age and gender. However, when adjusting for additional determinants of YKL-40, we found a negative correlation instead, which is probably due to the fact that some of these determinants also correlate with each other. We found no significant correlation between WHR and YKL-40. Previously, we have found a positive correlation between YKL-40 levels and BMI in small groups of lean, obese and morbidly obese subjects (unpublished data). Other studies have not been able to document correlations between YKL-40 and BMI [14], [16], [17], [48]. However, higher YKL-40 levels have been found in morbidly obese patients compared to lean subjects [16], [49], and even higher levels are seen in morbidly obese patients with T2D[49]. One study found that YKL-40 levels decreased significantly after bariatric surgery [16], whereas another study found a significant reduction in YKL-40 only after diet-induced weight loss and not after weight loss obtained by bariatric surgery [49]. This could be explained by the lack of a significant decrease in WHR and a higher post-intervention BMI in the group undergoing surgery compared to the group with diet-induced weight loss [49]. YKL-40 mRNA and protein levels have been found to be up-regulated in visceral adipose tissue in obese T2D patients compared to obese patients with normal glucose tolerance and lean subjects [49].

In the MONICA-10 population, we demonstrated that individuals presenting with minor allele homozygosity of rs12123883 had more than 40% higher triglyceride levels compared to individuals with either major allele homozygosity or heterozygosity. Similarly, individuals with minor allele homozygosity also had a more than twice as high prevalence of low HDL and a significantly higher median YKL-40 level. This could indicate that YKL-40 is expressed in higher concentrations in some genotypes, and could influence the development of dyslipidemia. However, only 13 participants (0.5%) were homozygote for the minor allele of rs12123883, which could indicate that the findings were random, and, in addition, we were not able to replicate the findings of higher triglyceride levels of minor allele homozygosity of rs12123883 in the Inter99 population. Plasma YKL-40 levels were not measured in the Inter99 population, which makes us unable to assess, whether the lack of a difference in lipid levels within the different genotypes is due to a lack of difference in YKL-40 levels. We found rather large discrepancies regarding the lipid levels between the two populations which may be due to the fact that different methods of analyses were used [24], [27], [28]. The variations of the lipid levels within each population were rather small, and the observed differences could be attributed to chance.

Several studies on SNPs of the YKL-40 encoding gene, *CHI3L1*, have documented that genetic variations of *CHI3L1* have an impact on circulating YKL-40-levels, both in healthy adults [18], [19] as well as in individuals with asthma[20], sarcoidosis[21], rheumatoid arthritis[18] and CAD[19]. It has also been hypothesized that variations of *CHI3L1* could even be associated with the development of CVD, but this is yet to be documented [19]. A study of 290 Koreans has shown significant associations between a promoter SNP of *CHI3L1* (rs946261) and levels of serum LDL, a major risk factor for the development of atherosclerosis. It was found that heterozygotes and major allele homozygotes of rs946261 had significantly higher LDL concentrations compared to minor allele homozygotes[22]. Unfortunately our analysis did not include rs946261. However, it is difficult to draw direct comparisons between Asian and European populations. Firstly, the alleles are not distributed equally in the two populations (http://www.ncbi.nlm.nih.gov/projects/SNP/snp_ref.cgi?rs=946261). Secondly, the lipid profile differs[50], [51], partly because of a difference in dietary habits, but perhaps also due to other regulatory mechanisms, such as hepatic lipase, with an impact on LDL levels[50].We were not able to demonstrate an association between any of the 12 SNPs we investigated and LDL levels.

Even though we found associations between YKL-40 levels and lipid levels, and between SNPs of *CHI3L1* and YKL-40 levels, we did not find convincing or reproducible associations between SNPs of *CHI3L1* and lipid levels, as would be expected if there were a causal link between elevated YKL-40 levels and the development of dyslipidemia. The most plausible explanation for this is that the positive associations between YKL-40 and lipid levels are not in fact causal. The finding of the positive associations is perhaps due to confounders or reverse causation and not to a direct influence of YKL-40 on the development of dyslipidemia. The high levels of YKL-40 found in individuals with elements of dyslipidemia and obesity could be caused by other factors, such as a larger burden of low grade inflammation in these individuals. Another explanation for the strong associations between YKL-40- and lipid levels but lack of associations between SNPs of *CHI3L1* and lipid levels could be that the SNPs investigated were not the functional SNPs directly determining the YKL-40 levels. Thus, further studies are needed to address this particular issue. Finally, we have to consider the size of our population as an explanation for the missing effect of variations of *CHI3L1* on the lipid levels. Perhaps it would be possible to document an effect with a larger population.

This is a cross sectional study regarding YKL-40 and lipids, which makes it difficult to differentiate, whether the observed associations are a matter of cause and effect or just coincidental associations. Secondly, one of the problems with genetic association studies is the risk of type 1 errors [52], which may explain the significant correlations between the SNPs of *CHI3L1* and lipid levels in the MONICA-10 population. To address this issue, we sought to replicate the findings in a similar population from the Inter99 study. However, it is a general problem with this type of study that positive results in one population turn out to be non-reproducible in the replication population, regardless of how well matched these populations may be[52].

In conclusion, we found a positive correlation of YKL-40 levels with triglyceride- and total cholesterol levels. We also found associations between certain SNPs of the YKL-40 encoding gene, *CHI3L1*, and serum YKL-40 levels. Positive associations between SNPs of *CHI3L1* and lipid levels were found, but could not be replicated in a similar but larger population, and therefore we cannot make any conclusions about the possible role of YKL-40 in the development of dyslipidemia. Further studies are needed to elucidate this.
